# Supplementary material for: Association between Trypanosoma cruzi DTU TcII and chronic Chagas disease clinical presentation and outcome in an urban cohort in Brazil
Source: PLoS One. 2020 Dec 2;15(12):e0243008. doi: 10.1371/journal.pone.0243008 (PMC7710061; doi:10.1371/journal.pone.0243008)
Supplement: S1 Table — (DOCX) [file pone.0243008.s001.docx]

S1 Table. Patients list with positive blood cultures samples, age, gender, transmission mode, serological results, DTUs, geographical origin and coordinates.

| **Positive Blood Cultures** | | | | | | | | | |
| --- | --- | --- | --- | --- | --- | --- | --- | --- | --- |
| Sample | Gender | Age | Transmission | IIF 1: | ELISA (RI) | DTU | City | State | Geographic coordinates |
| EMT1 | M | 36 | V | 640 | 7,7 | Tc II | Pilar | AL | 9° 35′ 50″ S, 35° 57′ 24″ W |
| EMT2(a,b,c) | F | 79 | V | 1280 | 8,2 | Tc II | Cachoeira | BA | 12° 37′ 4″ S, 38° 57′ 21″ W |
| EMT3 | F | 64 | V | 640 | 8,3 | Tc II | Conde | BA | 11° 48′ 50″ S, 37° 36′ 39″ W |
| EMT4(a,b,c) | F | 26 | V | 1280 | 8,4 | Tc VI | Barreiras | BA | 12° 9′ 10″ S, 44° 59′ 24″ W |
| EMT5(a,b) | F | 61 | V | 1280 | 8,3 | Tc II | Miguel Calmon | BA | 11° 25′ 44″ S, 40° 35′ 42″ W |
| EMT6(a,b) | M | 42 | Unknow | 160 | 7,3 | Tc II | Recife | PE | 8° 3′ 14″ S, 34° 52′ 51″ W |
| EMT7(a,b,c) | M | 46 | V | 640 | 7,8 | Tc II | Sta. Cruz de la Sierra | Bolívia | 17° 48′ 0″ S, 63° 10′ 0″ W |
| EMT8 | F | 56 | V | 320 | 7,4 | Tc II | Araçuaí | MG | 16° 51′ 0″ S, 42° 4′ 12″ W |
| EMT9 | F | 47 | V | 320 | 8,6 | Tc II | Laranjeiras | SE | 10° 48′ 22″ S, 37° 10′ 18″ W |
| EMT10 | F | 56 | V | 1280 | 8,2 | Tc II | S.Francisco do Conde | BA | 12° 37′ 40″ S, 38° 40′ 48″ W |
| EMT11 | F | 77 | V | 160 | 8,1 | Tc II | Cachoeira | BA | 12° 37′ 4″ S, 38° 57′ 21″ W |
| EMT12 | F | 71 | V | 320 | 7,9 | Tc II | Aliança | PE | 7° 36′ 10″ S, 35° 13′ 51″ W |
| EMT13 | F | 50 | V | 320 | 8,7 | Tc II | Araçoiaba | PE | 7° 47′ 24″ S, 35° 5′ 27″ W |
| EMT14 | F | 62 | V | 160 | 7,2 | Tc II | Itabaiana | PB | 7° 19′ 44″ S, 35° 19′ 58″ W |
| EMT15 | M | 63 | V | 1280 | 8,8 | Tc II | Machados | PE | 7° 41′ 9″ S, 35° 30′ 54″ W |
| EMT16 | F | 63 | V | 320 | 8,3 | Tc II | São Félix | BA | 12° 36′ 24.91″ S, 38° 58′ 9.82″ W |
| EMT17(a,b) | F | 37 | V | 160 | 8,3 | Tc II | Desterro | PB | 7° 17′ 27″ S, 37° 5′ 38″ W |
| EMT18 | F | 52 | C | 640 | 5,3 | Tc II | Cachoeira do Sul* | RS* | 30° 2′ 20″ S, 52° 53′ 38″ W* |
| EMT19(a,b) | F | 52 | Unknow | 640 | 7,6 | Tc II | João Pessoa | PB | 7° 5′ 0″ S, 34° 50′ 0″ W |
| EMT20(a,b) | F | 52 | V | 320 | 8,2 | Tc II | Teófilo Otoni | MG | 17° 51′ 28″ S, 41° 30′ 18″ W |
| EMT21 | F | 45 | T | 640 | 5,9 | Tc II | Macedônia | SP | 20° 8′ 45″ S, 50° 11′ 38″ W |
| EMT22(a,b) | F | 58 | V | 640 | 8,8 | Tc II | Feira do Santana | BA | 12° 16′ 1″ S, 38° 58′ 1″ W |
| EMT23 | F | 64 | V | 160 | 7,2 | Tc II | Cachoeira | BA | 12° 37′ 4″ S, 38° 57′ 21″ W |
| EMT24 | F | 24 | V | 640 | 8,3 | Tc II | Corumbá | MS | 19° 0′ 32″ S, 57° 39′ 10″ W |
| EMT25(a,b) | M | 60 | V | 640 | 8,2 | Tc II | Itambé | PE | 7° 24′ 36″ S, 35° 6′ 46″ W |
| EMT26(a,b,c) | M | 36 | V | 640 | 8,2 | Tc II | Wanderley | BA | 12° 7′ 12″ S, 43° 53′ 16″ W |
| EMT27 | F | 79 | V | 1280 | 8,6 | Tc II | Engenheiro Navarro | MG | 17° 16′ 48″ S, 43° 57′ 0″ W |
| EMT28(a,b) | M | 54 | V | 640 | 7 | Tc II | São José do Egito | PE | 7° 28′ 44″ S, 37° 16′ 28″ W |
| EMT29(a,b) | M | 64 | V | 640 | 8,8 | Tc VI | Guimarania | MG | 18° 50′ 38″ S, 46° 47′ 34″ W |
| EMT30 | F | 35 | V | 320 | 7,2 | Tc II | Campo Formoso | BA | 10° 30′ 32″ S, 40° 19′ 15″ W |
| EMT31(a,b) | M | 59 | V | 320 | 8 | Tc II | Timbaúba | PE | 7° 30′ 18″ S, 35° 19′ 4″ W |
| EMT32(a,b) | F | 55 | V | 640 | 8,7 | Tc II | Novo Cruzeiro | MG | 17° 28′ 4″ S, 41° 52′ 30″ W |
| EMT33 | M | 51 | V | 160 | 8,4 | Tc II | Novo Cruzeiro | MG | 17° 28′ 4″ S, 41° 52′ 30″ W |
| EMT34 | F | 39 | V | 640 | 8,7 | Tc II | Afogados da Ingazeira | PE | 7° 45′ 3″ S, 37° 38′ 20″ W |
| EMT35 | M | 42 | V | 1280 | 4,5 | Tc II | Taperoá | PB | 7° 12′ 23″ S, 36° 49′ 25″ W |
| EMT36(a,b) | F | 31 | V | 160 | 3,2 | ND | Serra Dourada | BA | 12° 45′ 39″ S, 43° 57′ 0″ W |
| EMT37(a,b,c) | M | 49 | V | 640 | 3,3 | Tc II | Campo Formoso | BA | 10° 30′ 32″ S, 40° 19′ 15″ W |
| EMT38(a,b) | F | 45 | V | 1280 | 3,5 | Tc II | Sertânia | PE | 8° 4′ 14″ S, 37° 15′ 57″ W |
| EMT39(a,b) | F | 56 | V | 320 | 2,6 | Tc II | Mundo Novo | BA | 11° 51′ 32″ S, 40° 28′ 19″ W |
| EMT40 | F | 62 | V | 640 | 3,9 | Tc II | Pinhão | SE | 10° 34′ 1″ S, 37° 43′ 22″ W |
| EMT41 | F | 61 | V | 1280 | 4,5 | Tc II | Timbaúba | PE | 7° 30′ 18″ S, 35° 19′ 4″ W |
| EMT42 | F | 40 | V | 640 | 3,3 | Tc II | Sertânia | PE | 8° 4′ 14″ S, 37° 15′ 57″ W |
| EMT43 | F | 53 | V | 640 | 6,7 | Tc II | Pedras de Fogo | PB | 7° 24′ 7″ S, 35° 6′ 57″ W |

V: vector borne; C: congenital; T: blood transfusion; IIF: indirect immunofluorescence; RI: reactivity índex; * for the case of congenital transmission, was located according to his mother place of birth.
