# Supplementary material for: Association between Trypanosoma cruzi DTU TcII and chronic Chagas disease clinical presentation and outcome in an urban cohort in Brazil
Source: PLoS One. 2020 Dec 2;15(12):e0243008. doi: 10.1371/journal.pone.0243008 (PMC7710061; doi:10.1371/journal.pone.0243008)

Figure 2 A. SL-IRac target

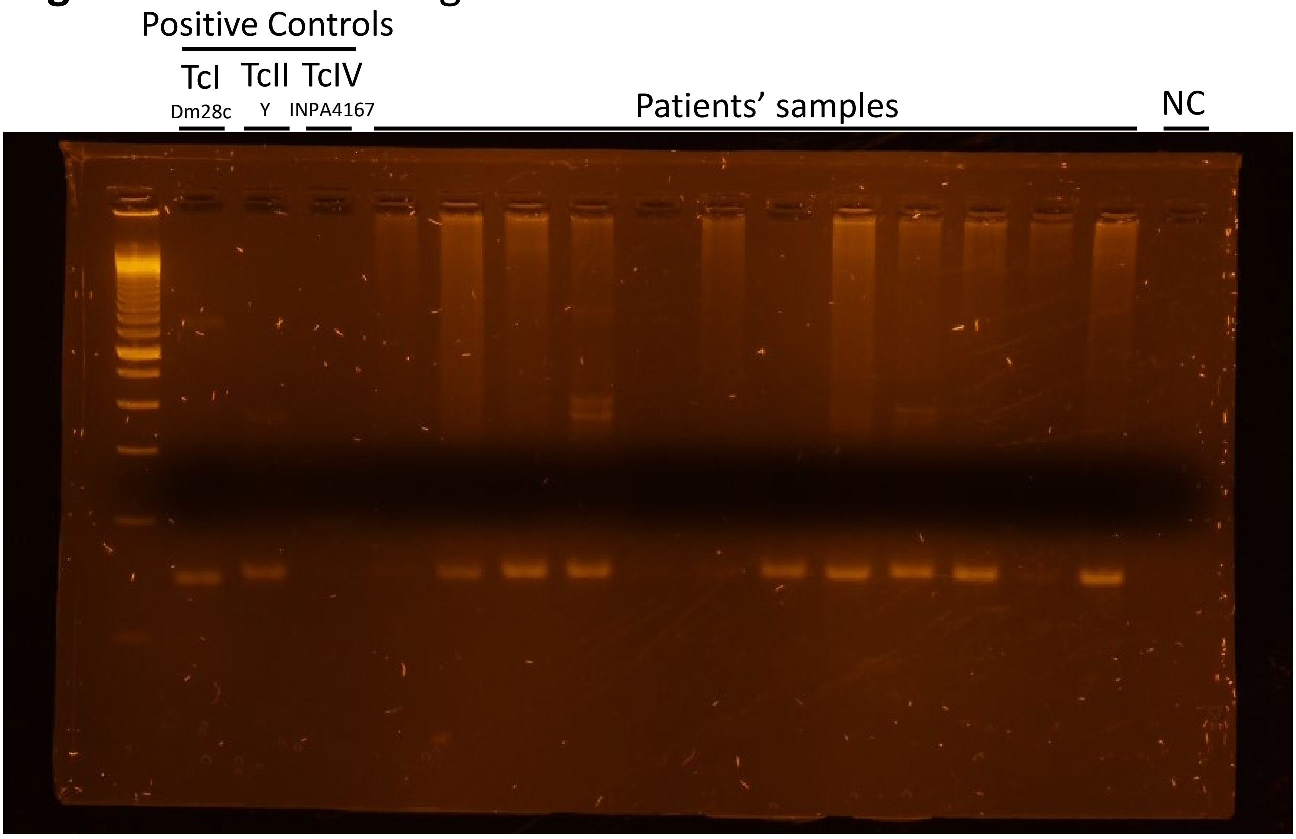

Figure 2 B. SL-IR I and II targets

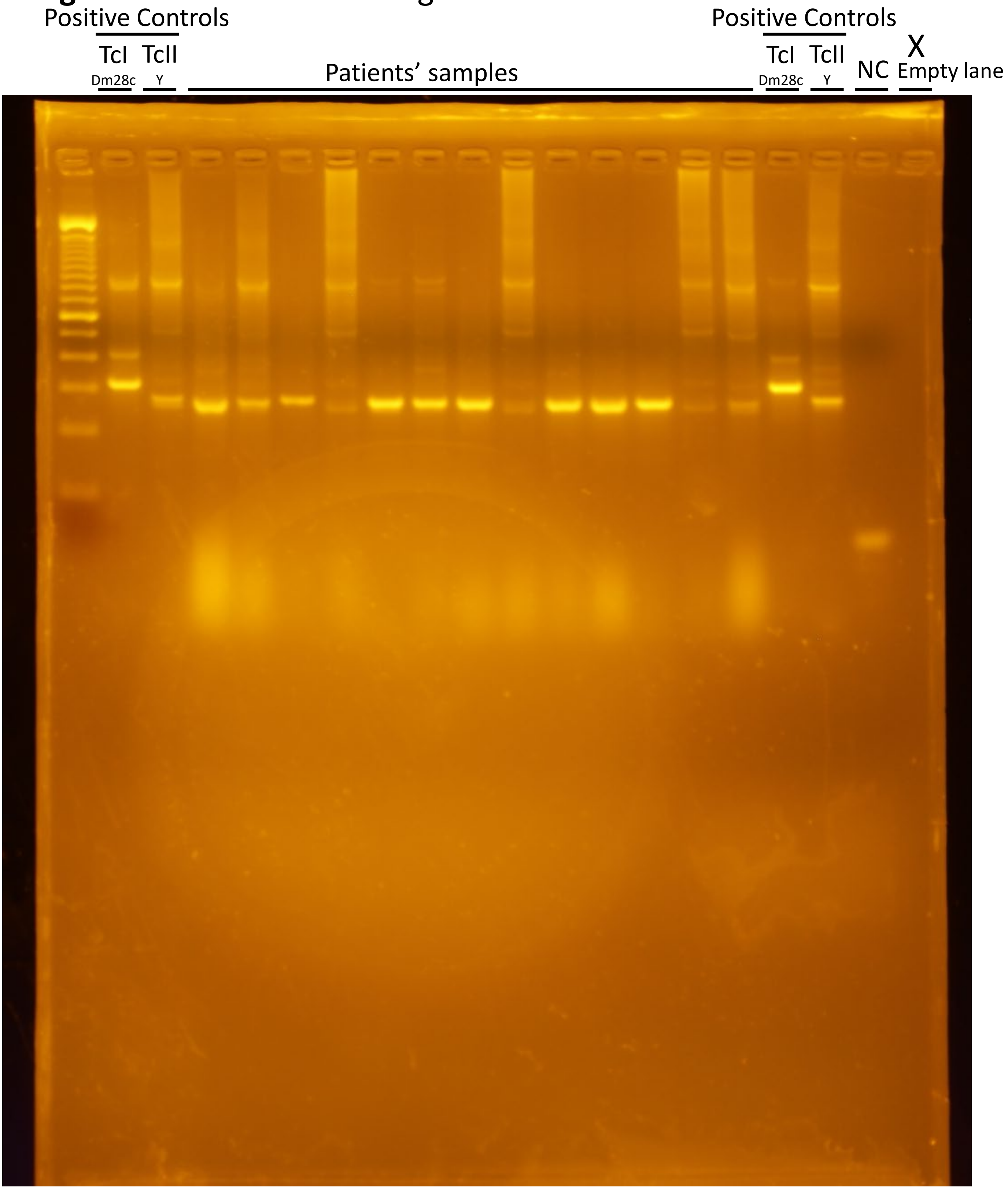

Figure 2 C. 24Sα rDNA target

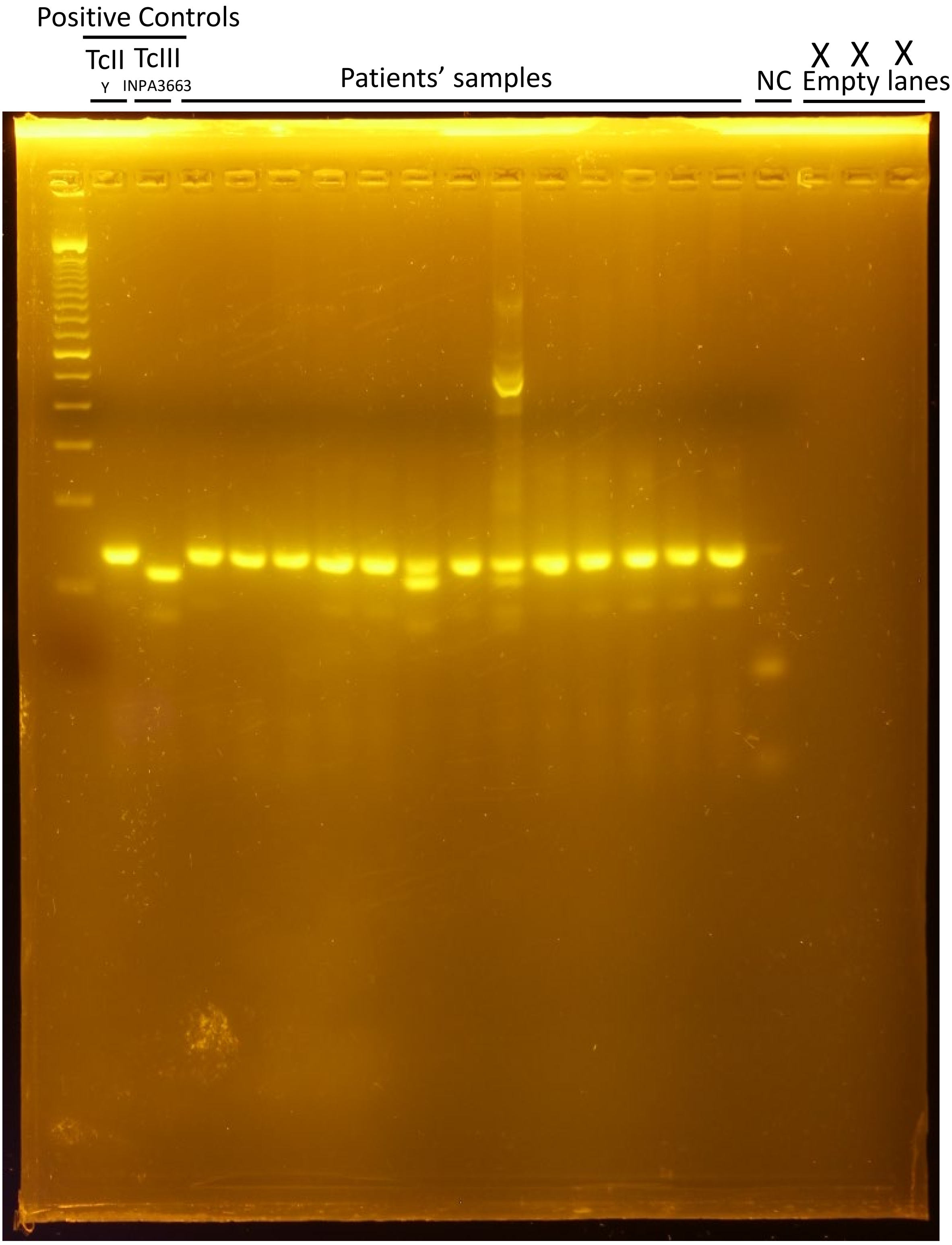

Figure 2 D. A10 target

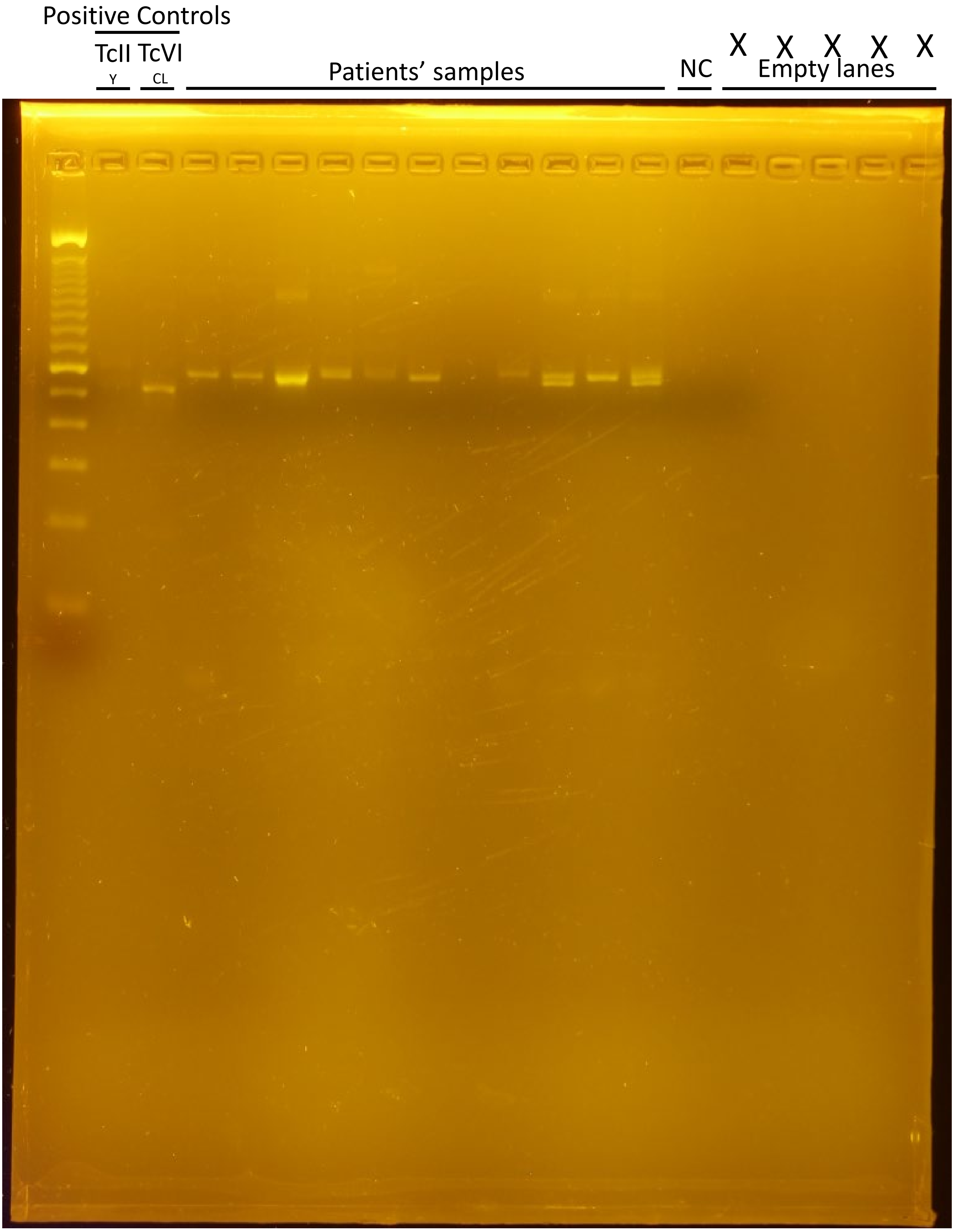

Supplement: S1 Raw images — (PDF) [file pone.0243008.s002.pdf]
