# Supplementary material for: Association between Trypanosoma cruzi DTU TcII and chronic Chagas disease clinical presentation and outcome in an urban cohort in Brazil
Source: PLoS One. 2020 Dec 2;15(12):e0243008. doi: 10.1371/journal.pone.0243008 (PMC7710061; doi:10.1371/journal.pone.0243008)
Supplement: Attachment — Submitted filename: response to reviewers PONE-D-2-26324R2.docx [file pone.0243008.s004.docx]

Rio de Janeiro, November 10^th^, 2020

Claudia Patricia Herrera, Ph.D.

Academic Editor

PLOS ONE

RE: Ref.: Ms. No. PONE-D-20-26324R1

MS TITLE: **Association between *Trypanosoma cruzi* DTU TcII and chronic Chagas disease clinical presentation and outcome in an urban cohort in Brazil.**

Dear Dr Herrera,

We are grateful to the editor’s recommendation on our paper. We addressed the further requirements as can be found below. All changes to the article are highlighted with track changes. A marked-up and an unmarked version of our revised paper were uploaded, as requested.

Sincerely,

Roberto Magalhães Saraiva, MD Ph.D

Instituto Nacional de Infectologia Evandro Chagas/Fundação Oswaldo Cruz

Av. Brasil, 4365

Rio de Janeiro, RJ, Brazil, 21040-360

E-mail: [roberto.saraiva@ini.fiocruz.br](mailto:roberto.saraiva@ini.fiocruz.br)

Editor Comments:

The authors well addressed the reviewers' comments. For the final acceptance of the article, I'm asking authors make a couple of minor edits on page 22, line 434, change "Limitations" by "Study limitations," and on page 18, line 349, replace "in the present article" with " in this study. "

**We made the requested changes. Please, see page 16 lines 294 to 295; and page 19, line 366 of the revised manuscript.**
